# hucMSC-Ex Alleviates IBD-Associated Intestinal Fibrosis by Inhibiting ERK Phosphorylation in Intestinal Fibroblasts

**DOI:** 10.1155/2023/2828981

**Published:** 2023-02-17

**Authors:** Yifei Wang, Yaqin Zhang, Bing Lu, Jianbo Xi, Dickson Kofi Wiredu Ocansey, Fei Mao, Donglin Hao, Yongmin Yan

**Affiliations:** ^1^Wujin Institute of Molecular Diagnostics and Precision Cancer Medicine of Jiangsu University, Jiangsu University, 213017 Changzhou, China; ^2^Key Laboratory of Medical Science and Laboratory Medicine of Jiangsu Province, School of Medicine, Jiangsu University, 212013 Zhenjiang, China; ^3^Zhenjiang College, 212028 Zhenjiang, China; ^4^Changzhou Key Laboratory of Molecular Diagnostics and Precision Cancer Medicine, 213017 Changzhou, China; ^5^Department of Laboratory Medicine, Wujin Hospital Affiliated with Jiangsu University (Wujin Clinical College of Xuzhou Medical University), Jiangsu University, 213017 Changzhou, China

## Abstract

**Background:**

Intestinal fibrosis, one of the complications of inflammatory bowel disease (IBD), is associated with fistula and intestinal stricture formation. There are currently no treatments for fibrosis. Mesenchymal stem cell-derived exosomes have been proven to exert inhibitory and reversal effects in IBD and other organ fibrosis. In this study, we explored the role of human umbilical cord mesenchymal stem cell-derived exosomes (hucMSC-Ex) in IBD-related fibrosis and its associated mechanism to provide new ideas for the prevention and treatment of IBD-related intestinal fibrosis.

**Methods:**

We established a DSS-induced mouse IBD-related intestinal fibrosis model and observed the effect of hucMSC-Ex on the mouse model. We also used the TGF-induced human intestinal fibroblast CCD-18Co to observe the role of hucMSC-Ex in the proliferation, migration, and activation of intestinal fibroblasts. Having observed that the extracellular-signal-regulated kinase (ERK) pathway in intestinal fibrosis can be inhibited by hucMSC-Ex, we treated intestinal fibroblasts with an ERK inhibitor to emphasize the potential target of ERK phosphorylation in the treatment of IBD-associated intestinal fibrosis.

**Results:**

In the animal model of IBD-related fibrosis, hucMSC-Ex alleviated inflammation-related fibrosis as evident in the thinning of the mice's intestinal wall and decreased expression of related molecules. Moreover, hucMSC-Ex inhibited TGF-*β*-induced proliferation, migration, and activation of human intestinal fibroblasts, and ERK phosphorylation played a key role in IBD-associated fibrosis. The inhibition of ERK decreased the expression of fibrosis-related indicators such as *α*-SMA, fibronectin, and collagen I.

**Conclusion:**

hucMSC-Ex alleviates DSS-induced IBD-related intestinal fibrosis by inhibiting profibrotic molecules and intestinal fibroblast proliferation and migration by decreasing ERK phosphorylation.

## 1. Introduction

Inflammation bowel disease (IBD) is a severe intestinal inflammation, including ulcerative colitis (UC) and Crohn's disease (CD). Intestinal fibrosis is a complication in IBD, especially in CD, which triggers the occurrence of stricture in the locations involved. Apart from stricture in the intestinal lumen, IBD-related fibrosis can induce anal fistula, an important complication in CD, when the inflammation appears in the rectum [1].

The mechanism of intestinal fibrosis is not completely confirmed. In a recent study, it is widely accepted that transforming growth factor-*β* (TGF-*β*) is a key molecule in the progression of intestinal fibrosis. TGF-*β* functions in fibrosis by influencing downstream signaling pathways such as the TGF-*β*/smad pathway and the wnt/*β*-catenin pathway [2]. Several cells are involved in the development of intestinal fibrosis, including fibroblasts, epithelial cells, and endothelial cells. Most notably, fibroblasts play a crucial role. Under the stimulation of factors related to the development of fibrosis, fibroblasts can be activated with increased proliferative ability, thus promoting the occurrence of intestinal fibrosis. Under normal conditions, fibroblasts produce an extracellular matrix (ECM) to form the structural framework of the tissue. However, when inflammation and cell necrosis and apoptosis occur in the tissue, a large number of cytokines that promote the occurrence of fibrosis are increasingly produced. These factors promote the local activation of fibroblasts, and a large number of ECM are generated at the same time, resulting in the occurrence of intestinal fibrosis [3].

In addition to not fully understanding the mechanism involved in intestinal fibrosis, there is also no ideal therapeutic method. Although some of the existing anti-inflammation drugs have been shown to prevent fibrosis to a certain extent, those therapies are not always useful as antifibrotic. Mesenchymal stem cells (MSCs) have the ability to self-renew and multidifferentiate [4]. There are pieces of evidence that MSCs play important therapeutic roles in IBD [5] and could relieve or reverse fibrosis, as trial results of clinical patients showed that allogeneic adipose-derived MSCs could reduce the occurrence of fistulas (which are closely related to the occurrence of fibrosis) in CD patients [6]. Bone marrow-derived MSCs reduced fibrotic-associated activities such as collagen deposition and EMT in the TNBS-induced colitis mouse model [7], while adipose-derived MSCs mediated the downregulation of fibrogenesis via controlling ECM turnover through the induction of a decreased expression of profibrotic proteins and genes by releasing hepatocyte growth factor (HGF) and tumor necrosis factor-stimulated gene 6 (TSG-6) in colorectal fibrosis [8].

Exosomes are small vesicles secreted by cells and are considered to be a type of extracellular vesicle (EV). They have a diameter between 30 and 150 nm and a density between 1.13 and 1.19 g/ml [9]. Just like MSCs, several studies have demonstrated that MSC-derived exosomes play a role in easing IBD [10]. For example, by using human umbilical cord mesenchymal stem cell-derived exosomes (hucMSC-Ex) in the DSS-induced intestinal fibrosis model, the authors found that hucMSC-Ex regulated the occurrence of inflammation and relieved enteritis by reducing the levels of TNF-*α* and IL-1*β* and increasing the level of IL-10 [11]. Moreover, MSC-derived exosomes play a role in the fibrosis of organs, including intestinal fibrosis, liver fibrosis, pulmonary fibrosis, and kidney fibrosis [12]. Regardless, the exact role of hucMSC-Ex in the occurrence and development of intestinal fibrosis and the associated therapeutic potential remain unexplored.

Therefore, in this study, we explored the role and mechanism of hucMSC-Ex in mitigating IBD-related intestinal fibrosis and provide new insights into intestinal fibrosis treatment approaches.

## 2. Methods

The study was approved by the Ethical Committee of Jiangsu University (2012258).

### 2.1. Isolation of hucMSCs

Fresh umbilical cords were obtained from the Zhenjiang Fourth People's Hospital. The umbilical cords were washed in PBS for 30 minutes and were then cut into pieces and placed in clean 3.5 cm small dishes to adhere to the wall. After incubation at 37°C in a 5% CO_2_ environment, a small amount of *α*-MEM (Invitrogen) containing 15% FBS (FBS; Excel) and 5% penicillin-streptomycin (Invitrogen) was used to cover segments in dishes, and the solution was replaced every 3 days. When observation under the microscope revealed long spindle cells near the small pieces of tissues and fused to 80%, it indicates that hucMSCs could be digested.

### 2.2. Cell Culture

hucMSCs were isolated and cultured in *α*-MEM with 10% fetal bovine serum (FBS; Excel) and 1% penicillin-streptomycin (Invitrogen). When the hucMSCs in the Petri dish grew to about 70% confluence, the nutrient solution was replaced by a new *α*-MEM (Invitrogen) nutrient solution, which is made up of 10% exosome-free FBS and 5% penicillin-streptomycin (Invitrogen). After culturing in the incubator for 48 hours, the supernatant was collected and the above process was repeated until the P7 passage. Human intestinal fibroblasts CCD-18Co were purchased from Beiner Biotechnology and cultured in RPMI 1640 medium (Invitrogen), which constitutes 10% FBS (FBS; Excel) and 5% penicillin-streptomycin (Invitrogen).

### 2.3. Exosome Extraction

hucMSC-Ex was extracted by ultracentrifugation. The supernatant of hucMSC culture was centrifuged at 4°C, 2000g for 30 minutes. The supernatant of the previous step was centrifuged at 4°C, 10000g for 30 minutes. Next, the obtained supernatant was concentrated to 100 ml using 100KD ultrafiltration tubes. The concentrated liquid was further centrifuged at 4°C, 100,000g for 70 minutes. The resultant precipitate was saved, and an appropriate amount of PBS was added to dissolve it. The dissolved solution was centrifuged at 4°C, 100,000g for 70 minutes, the precipitate was collected, and an appropriate amount of PBS was added to dissolve the precipitate. Finally, the resultant solution was filtered with a 0.22 *μ*m filter and distributed into EP tubes for storage in a -80°C refrigerator.

### 2.4. NanoSight Nanoparticle Tracking Analysis (NTA)

The standard solution was used to generate a standard curve, and diluted hucMSC-Ex was added to the sample chamber and read the data. Use the software for image capture and analysis.

### 2.5. TEM Scanning

About 20 *μ*l hucMSC-Ex solution was dripped onto a 2 mm copper mesh. The copper mesh was allowed to stand, and excess liquid was wiped off the edge. hucMSC-Ex was counterstained with 3% phosphotungstic acid, and pictures were taken with the transmission electron microscope after drying.

### 2.6. Animal Model

The study used 6-8-week-old female C57BL/6 mice purchased from the Animal Experiment Center of Jiangsu University. The mice were weighed and randomly divided into three groups: NC group, DSS group, and hucMSC-Ex group (Sup. 1A). The NC group was fed normally and given double-distilled water (ddH_2_O), while the DSS group was given 2% DSS solution throughout the first week and two weeks later and given food for three cycles and ddH_2_O when 2% DSS solution is not being administered. The hucMSC-Ex group received 100 *μ*l (1 × 10^9^ particles) hucMSC-Ex by tail vein injection on the 3rd, 10th, 17th, 24th, 31st, 38th, 45th, 52nd, and 59th days. All mice were weighed every 3 days, and fecal appearance and consistency were observed. On the 63rd day, the mice were sacrificed.

### 2.7. DAI Scores

The disease activity index (DAI) was calculated based on the criteria shown in Table 1.

### 2.8. Staining

Staining kits (Solarbio) were used for hematoxylin and eosin (H&E), Masson, and Sirius red staining of paraffin sections of mouse intestinal tissues.

### 2.9. Western Blot

A moderate volume of RIPA solution was added to cells and mixed thoroughly. The solution was shaken on a vortex shaker for 1 minute and allowed to stand on ice for 5 minutes. The process was repeated 5 times in total. The solution was centrifuged at 4°C, 12,000g for 15 minutes to obtain the supernatant, which is the protein solution. SDS-PAGE electrophoresis was used to separate target proteins and transfer them to a PVDF membrane. The PVDF membranes were soaked in 5% skim milk to block nonspecific proteins. The membranes were incubated with corresponding primary antibodies: anti-CD9 (1 : 500; Proteintech), anti-CD81 (1 : 500; Proteintech), anti-HSP70 (1 : 500; Affinity), anti-calnexin (1 : 500; Affinity), anti-*β*-actin (1 : 500; ABclonal), anti-*α*-SMA (1 : 500; Proteintech), anti-collagen I (1 : 500; Abcam), anti-collagen III (1 : 500; Proteintech), anti-fibronectin (1 : 500; Proteintech), and anti-t-ERK (1 : 800; Abcam) overnight. After washing with 1x TBS/T buffer, the membranes were incubated with the secondary antibody (1 : 2000; Invitrogen) for 30 min at 37°C. Pictures were taken with a chemical gel imaging system (GE).

### 2.10. Quantitative Real-Time Polymerase Chain Reaction (QRT-PCR)

RNA was extracted by TRIzol (Vazyme) and dissolved in DEPC water. cDNA was obtained by using the Vazyme kit on the PCR machine (25°C, 5 minutes and 85°C, 5 seconds). Obtained cDNA was used to perform QRT-PCR using the Vazyme kit on Step One Plus Real-Time PCR System (ABI). The reaction process was 95°C, 5 minutes; 95°C, 30 seconds; 58~60°C (flexible setting according to different primers), 30 seconds; and 72°C, 30 seconds, for a total of 40 cycles. The specific system reagents and volumes are shown in Tables 2 and 3. Primer sequences used are presented in Table 4.

### 2.11. Immunochemistry (IHC)

Mouse colon tissues were fixed in 4% paraformaldehyde. The tissues were dehydrated, cleared, and embedded in paraffin, followed by microtome sectioning at the First People's Hospital of Zhenjiang City. The slices were kept at 56°C for 4 hours to prevent the tissue from falling off in subsequent procedures. The tissue slices were deparaffinized, and the sections were placed in a hydrogen oxide-methanol solution (1 : 9 mixed with 30% hydrogen peroxide and methanol) and kept at room temperature for 30 minutes. Slices were washed 3 times with histochemical PBS, immersed in citrate solution, and steamed in an electric oven for 30 minutes. After cooling, the histochemical PBS was washed 3 times, followed by blocking nonspecific proteins in sections with BSA for at least 30 minutes. The protein antibody (Proteintech) was diluted to 1 : 200 with 5% BSA and used to cover the tissue at 4°C overnight. It was then washed 3 times with PBS, incubated with the secondary antibody (1 : 300; Invitrogen) and SABC (1 : 300; Bio-Rad), and finally observed under the microscope after adding DAB (Bio-Rad), looking for golden yellow positive areas. The hematoxylin staining solution was used to counterstain the slide, and pictures were taken under the microscope.

### 2.12. CCK8

The TGF-*β*-induced cell models (Sup. 1B) were constructed in 96-well plates. The 96-well plates were placed in 37°C and 5% CO_2_ for 24 or 48 hours, followed by the addition of 10 *μ*l CCK8 and incubation at 37°C and 5% CO_2_ for 2 hours. A microplate reader was used to detect the absorbance of the solution in the 96-well plate at 450 nm.

### 2.13. Cell Clone

After the construction of the TGF-*β*-induced cell models (Sup. 1B) in 6-well plates, 15 ng/ml TGF-*β* or hucMSC-Ex was when all cells had adhered to the wall. The plates were kept at 37°C and 5% CO_2_. After 14 days, cells were fixed with 4% paraformaldehyde and stained with 0.5% crystal violet staining solution. The outcome was examined, and images were taken.

### 2.14. Transwell Migration Assay

The TGF-*β*-induced cell models (Sup. 1B) were constructed in 6-well plates and incubated at 37°C and 5% CO_2_ for 48 hours. The Transwell chamber was put into a 24-well plate, and 200 *μ*l of RIPA 1640 (Bioind) nutrient solution was added to the well. Three groups of cells in the six-well plate were digested and resuspended in FBS-free RPMI 1640 nutrient solution. A certain amount of resuspension was added to different Transwell wells, and the 24-well plate was cultured at 37°C and 5% CO_2_ for 18 hours. Cells were stained with 0.5% crystal violet, and images were obtained under the microscope.

### 2.15. Immunofluorescence (IF)

Cell slides were placed in a 24-well plate, and cells were added to construct a cell model induced by TGF-*β* (Sup. 1B). After the treatment, 4% paraformaldehyde was added to the wells for 30-60 minutes to fix the cells. Wells were washed with PBS 3 times, and 500 *μ*l 0.1% Triton-100 solution was added at room temperature for 30 minutes. PBS was used to wash 3 times, and 5% BSA was added to the slides for blocking nonspecific protein. The cells were incubated with corresponding primary antibodies: anti-*α*-SMA (1 : 500; Proteintech) and anti-fibronectin (1 : 500; Proteintech) overnight. A fluorescent secondary antibody (SAB) was added to the slide and incubate for 1 hour at 37°C in the dark and then washed with PBS 3 times. Nuclei were stained with Hoechst 33342, the slides were fixed with an antifluorescence quencher, and images were taken under the laser scanning confocal microscope.

### 2.16. Statistical Analysis

Data for all results were shown as mean ± standard error. Analysis of variance was performed by using the GraphPad Prism software, and *P* values of < 0.05 were considered statistically significant.

## 3. Results

### 3.1. Identification of hucMSC-Ex

**Figure 1:**
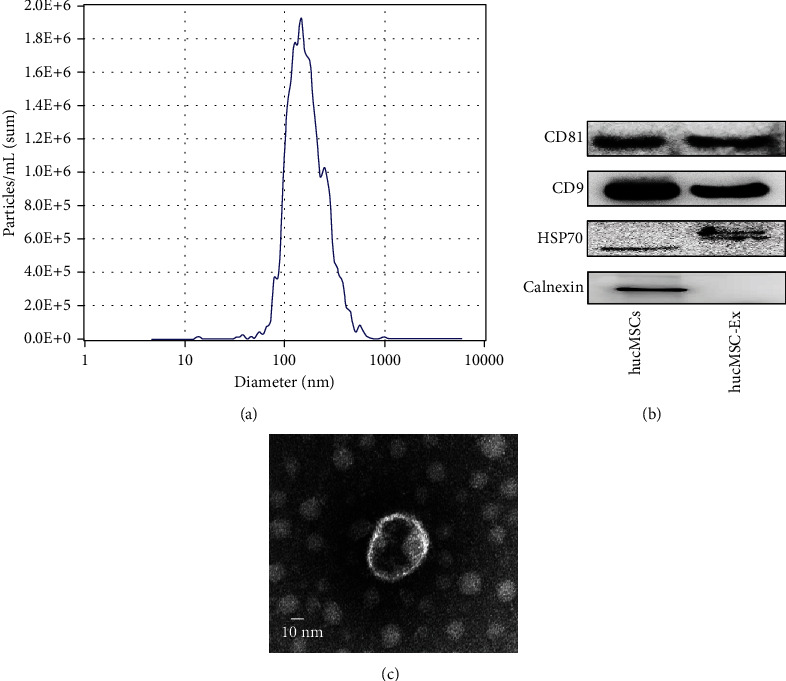
Identification of hucMSC-Ex. (a) The result of NanoSight nanoparticle tracking analyzer detection. (b) The morphology of hucMSC-Ex in transmission electron microscope. (c) Western blot analysis of hucMSC-Ex protein markers.

The extracted hucMSC-Ex were examined with nanoparticle tracking analysis (NTA), which revealed particle diameter between 50 and 150 nm (Figure 1(a)). Transmission electron microscopy (TEM) results showed vesicle-like particles (Figure 1(b)). Western blot results confirmed the presence of protein markers of hucMSC-Ex (CD9, CD81, and HSP70), using calnexin as a negative control marker (Figure 1(c)). The above results certified that the extracted sample was hucMSC-Ex. For subsequent utilization of the hucMSC-Ex, it was diluted to 1 × 10^10^/ml.

### 3.2. hucMSC-Ex Alleviates DSS-Induced IBD-Related Intestinal Fibrosis in Mice

**Figure 2:**
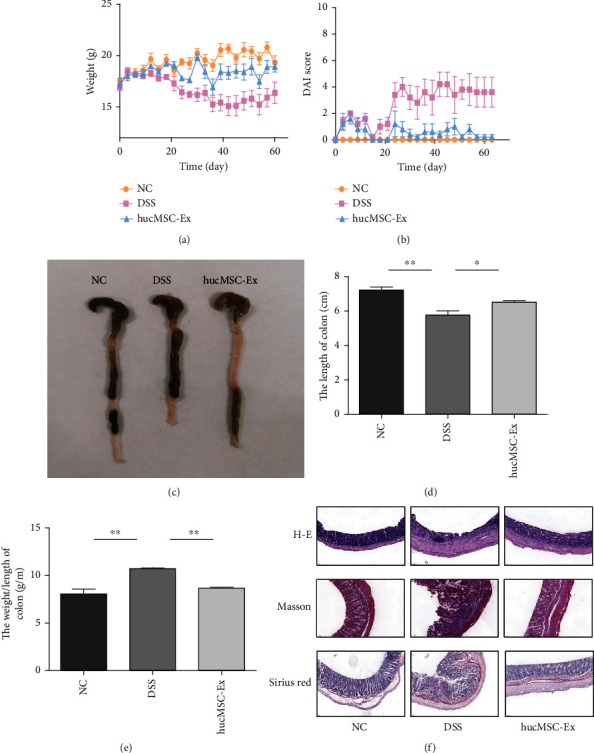
hucMSC-Ex alleviates DSS-induced IBD-related intestinal fibrosis in mice. (a) Weights of mice. (b) DAI scores of mice. (c) Colon appearance in mice. (d) Statistical chart of mice colon length, ^∗^*P* < 0.05 and ^∗∗^*P* < 0.01. (e) Weight/length ratio of mouse colon, ^∗∗^*P* < 0.01. (f) H&E, Sirius red, and Masson staining of mouse colon (200x).

To assess whether hucMSC-Ex could reduce or inhibit the development of IBD-related intestinal fibrosis, we constructed a DSS-induced mouse model consisting of a normal control (NC) group, a DSS group, and a hucMSC-Ex group. The hucMSC-Ex group received 100 *μ*l (1 × 10^9^ particles) of hucMSC-Ex via the tail vein on the third day of every week, starting from the first week. The results showed variations in the weight of the different groups. With time, the weight of mice in the NC group gradually increased while that of the DSS group showed a downward trend with the administration cycle. However, body weight loss was effectively suppressed after the hucMSC-Ex injection (Figure 2(a)). At the same time, the mice in the DSS group had relatively severe loose stools or even bloody stool and symptoms in each DSS administration cycle while the mice in the hucMSC-Ex group had mild symptoms (Figure 2(b)). On day 63, all the mice were sacrificed and colon tissues were taken for examination. It was found that the colon length of mice in the DSS group was shortened and the weight/length ratio increased, but hucMSC-Ex treatment significantly restored the colon length and decreased the weight/length ratio (Figures 2(c)–2(e)). To assess the degree of colitis-associated damage to colon tissue and the repair ability of hucMSC-Ex, H&E, Masson, and Sirius red staining were performed. The results of the H&E staining showed increased tissue damage and thickness of the colon of the DSS group while that of the hucMSC-Ex group improved. Moreover, the Masson staining and Sirius red staining showed that compared with the NC group, the collagen deposition in the DSS group was increased but decreased in the hucMSC-Ex group (Figure 2(f)).

### 3.3. hucMSC-Ex Inhibits the Expression of *α*-SMA, TGF-*β*, Collagen I, Collagen III, and Fibronectin in Mouse Colons

**Figure 3:**
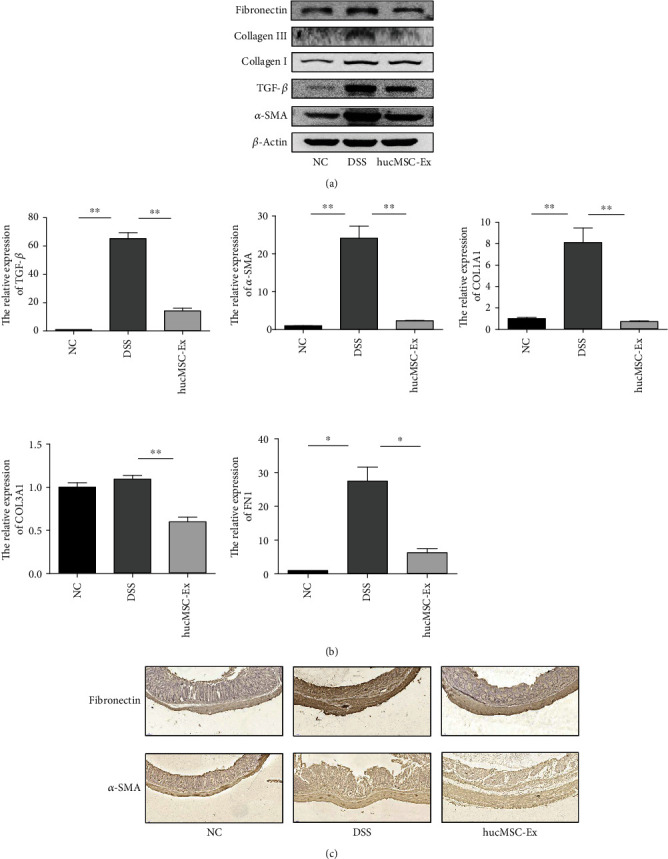
hucMSC-Ex inhibits the expression of *α*-SMA, TGF-*β*, collagen I, collagen III, and fibronectin in mouse colons. (a) Western blot analysis of *α*-SMA, TGF-*β*, collagen I, collagen III, and fibronectin expression in mouse colon. (b) QRT-PCR analysis of TGF-*β*, *α*-SMA, COL1A1, COL3A1, and FN1 expression in mouse colon tissue (^∗^*P* < 0.05 and ^∗∗^*P* < 0.01). (c) The expression levels of fibronectin and *α*-SMA in mouse colon tissue as detected by IHC (200x).

To observe the effect of hucMSC-Ex on fibrosis-related molecules in mice, we extracted protein and RNA from colon tissues for western blot and PCR, respectively, and performed IHC to observe the changes in mice under the influence of hucMSC-Ex. The results showed that compared with mice in the DSS group, the expression of fibronectin, collagen I and III, TGF-*β*, and *α*-SMA was down-regulated in the hucMSC-Ex group (Figure 3(a)). QRT-PCR analysis showed that the expression of TGF-*β*, *α*-SMA, COL1A1, COL3A1, and FN1 in the DSS group was increased but significantly decreased in the hucMSC-Ex group (Figure 3(b)). In addition, the results of IHC indicated that the expressions of fibronectin and *α*-SMA in the colon tissue of the DSS mice were significantly increased while the intervention of hucMSC-Ex inhibited the expression of FN but could not significantly change *α*-SMA (Figure 3(c)).

### 3.4. hucMSC-Ex Inhibits Human Intestinal Fibroblast Proliferation and Migration

**Figure 4:**
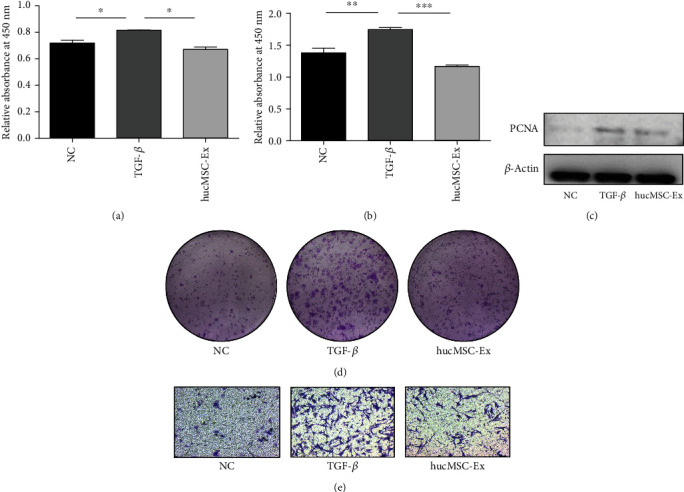
hucMSC-Ex inhibits human colonic fibroblast proliferation and migration. (a) CCK8 of intestinal fibroblasts after treatment for 24 hours, ^∗^*P* < 0.05. (b) CCK8 of intestinal fibroblasts after treatment for 48 hours, ^∗∗^*P* < 0.01 and ^∗∗∗^*P* < 0.0001. (c) Western blot analysis of PCNA expression in intestinal fibroblasts after treatment for 48 hours. (d) The results of cloning experiment of intestinal fibroblasts for 14 days. (e) Transwell assay of intestinal fibroblasts after 18 hours of treatment (200x).

We used TGF-*β* to induce the proliferation and migration of human colonic fibroblasts and observed the role of hucMSC-Ex in regulating these phenomena. CCK8 analysis was performed on the cells after treatment for 24 hours and 48 hours. The results showed that the proliferation of human colonic fibroblasts was enhanced under the stimulation of TGF-*β* but was significantly inhibited after the intervention of hucMSC-Ex (*P* < 0.05, Figures 4(a) and 4(b)). In addition, western blot analysis of the protein expression of fibroblast after 48 hours of treatment showed that proliferating cell nuclear antigen (PCNA) increased after TGF-*β* treatment and decreased after hucMSC-Ex intervention (Figure 4(c)). Furthermore, cell cloning experiments showed that the number of cell clusters in the TGF-*β* group increased after 14 days, while the proliferation ability in the hucMSC-Ex-treated group was reduced (Figure 4(d)). After 48 hours of treatment, the migration ability of fibroblasts in the TGF-*β* group was increased. In contrast, the migration ability of the cells in the hucMSC-Ex group was significantly inhibited (Figure 4(e)). All the above results indicate that hucMSC-Ex could inhibit the proliferation and migration of human intestinal fibroblasts.

### 3.5. hucMSC-Ex Inhibits the Expression of *α*-SMA, Collagen I, Collagen III, and Fibronectin in Intestinal Fibroblasts

**Figure 5:**
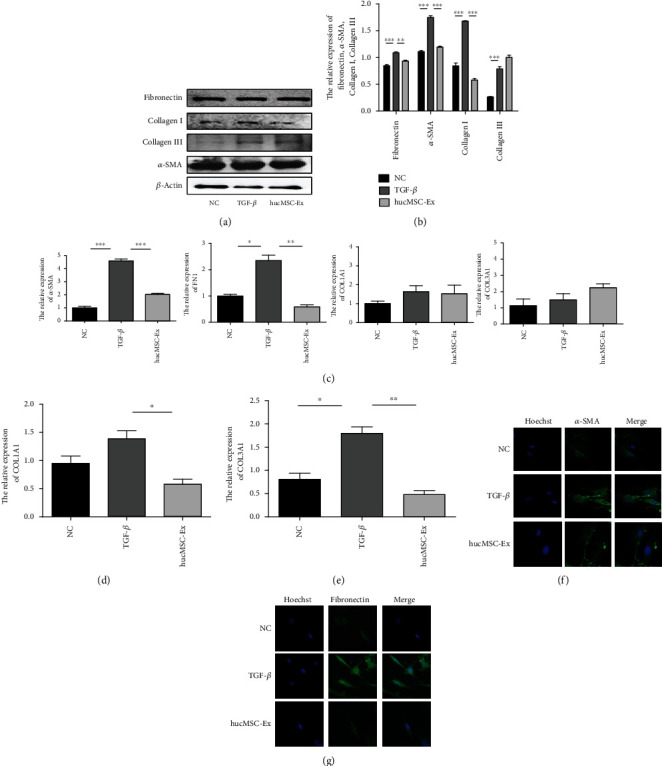
hucMSC-Ex inhibits the expression of *α*-SMA, collagen I, collagen III, and fibronectin in colonic fibroblasts. (a) Western blot analysis of *α*-SMA, collagen I, collagen III, and fibronectin expression in human intestinal fibroblasts CCD18-Co after treatment with TGF-*β* for 48 hours. (b) Grayscale scanning of (a), ^∗∗^*P* < 0.01 and ^∗∗∗^*P* < 0.001. (c) QPCR analysis of *α*-SMA, COL1A1, COL3A1, and FN1 expression in human intestinal fibroblasts CCD-18Co after treatment with TGF-*β* for 48 hours, ^∗^*P* < 0.05, ^∗∗^*P* < 0.01, and ^∗∗∗^*P* < 0.001. (d) QRT-PCR analysis of COL1A1 expression in human intestinal fibroblasts CCD-18Co after treatment with TGF-*β* for 24 hours, ^∗^*P* < 0.05. (e) QRT-PCR analysis of COL1A1 expression in human intestinal fibroblasts CCD-18Co after treatment with TGF-*β* for 72 hours, ^∗^*P* < 0.05 and ^∗∗^*P* < 0.01; (f) IF analysis of *α*-SMA expression in human intestinal fibroblasts CCD-18Co after treatment with TGF-*β* for 48 hours (400x). (g) IF analysis of fibronectin expression in human intestinal fibroblasts CCD-18Co after treatment with TGF-*β* for 48 hours (400x).

After demonstrating that hucMSC-Ex could inhibit the proliferation and migration of human colonic fibroblast CCD18-Co, we continued to investigate the role of hucMSC-Ex on some fibrosis-related molecules. After inducing the cells with TGF-*β*, the protein was extracted after 48 hours for western blot. The results showed that the fibroblast activation marker *α*-SMA increased in the TGF-*β* group, while inhibited in the hucMSC-Ex group. Meanwhile, the expressions of fibronectin and collagen I were increased after stimulation with TGF-*β* and decreased after treatment with hucMSC-Ex. However, compared with animal tissues, the expression of collagen III in the TGF-*β* group increased at 48 hours and continued to increase after the hucMSC-Ex treatment (Figures 5(a) and 5(b)). QRT-PCR results showed that fibroblasts treated for 48 hours had increased expression of *α*-SMA, COL1A1, and FN1 genes in the TGF-*β* group, and the expression in the hucMSC-Ex group was relatively decreased (*P* < 0.05). However, the expression of COL3A1 genes increased in the TGF-*β* group, and the changes were not obvious or even continued to increase after hucMSC-Ex treatment (Figure 5(c)). For fibroblasts treated for 24 hours, QRT-PCR showed that the expression of *α*-SMA and collagen I decreased significantly after hucMSC-Ex intervention (Figure 5(d)). Moreover, fibroblast treated for 72 hours showed significantly downregulated expression of collagen III after hucMSC-Ex intervention (Figure 5(e)). IF results showed that the expression of *α*-SMA and fibronectin decreased after hucMSC-Ex treatment (Figures 5(f) and 5(g)). All the above results indicated that hucMSC-Ex could inhibit the expression of *α*-SMA, collagen I, collagen III, and fibronectin in human colonic fibroblasts.

### 3.6. hucMSC-Ex Inhibits Fibrosis-Related Molecules by Inhibiting ERK Phosphorylation

**Figure 6:**
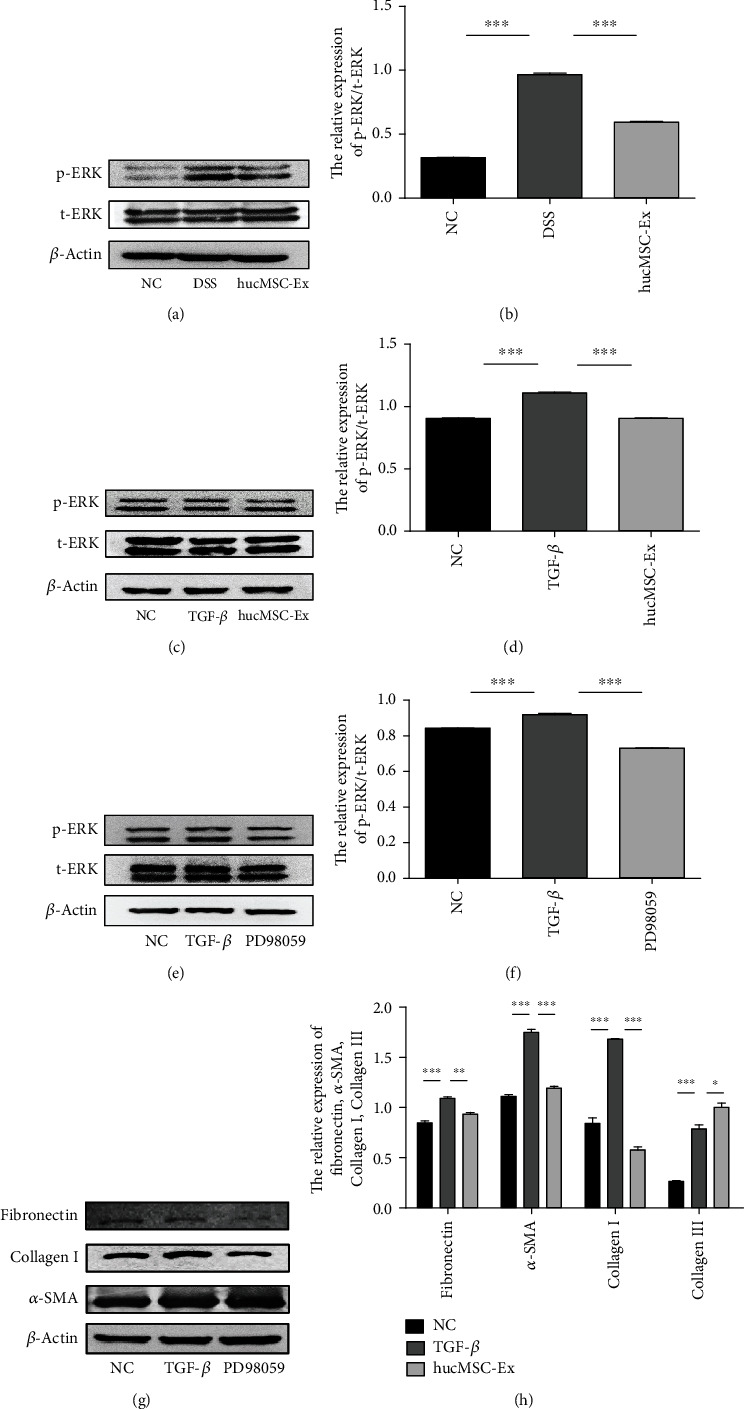
hucMSC-Ex inhibits the expression of fibrosis-related molecules by inhibiting ERK phosphorylation. (a) Western blot analysis of ERK expression in DSS-induced IBD-associated fibrosis in mice. (b) Grayscale scanning of (a), ^∗∗∗^*P* < 0.001. (c) Western blot analysis of ERK expression in human intestinal fibroblasts CCD-18Co after treatment with TGF-*β* or hucMSC-Ex for 48 hours. (d) Grayscale scanning of (c), ^∗∗∗^*P* < 0.001. (e) The expressions of t-ERK and p-ERK. (f) Grayscale scanning of (e), ^∗∗∗^*P* < 0.001. (g) The expressions of *α*-SMA, collagen I, and fibronectin. (h) Grayscale scanning of (g), ^∗∗^*P* < 0.01 and ^∗∗∗^*P* < 0.001.

Having demonstrated that hucMSC-Ex could alleviate inflammatory-related fibrosis in vivo and in vitro, we proceeded to examine the molecular mechanism or pathway involved. Western blot analysis of mouse colon tissue protein showed that the ratio of p-ERK/t-ERK in the DSS group mice was increased which indicates that ERK phosphorylated is increased in intestinal fibrosis but decreased after hucMSC-Ex treatment (Figures 6(a) and 6(b)). Moreover, western blot results of fibroblasts treated for 48 hours showed that the ratio of p-ERK/t-ERK was increased in the TGF-*β* group but decreased in the hucMSC-Ex group (Figures 6(c) and 6(d)). These observations presented ERK phosphorylation, which was increased in intestinal fibrosis and inhibited by hucMSC-Ex, as an interesting phenomenon for further exploration. Thus, the ERK inhibitor PD98059 was used to treat human intestinal fibroblasts (Sup. 1C). Fibroblasts treated for 48 hours showed downregulated expression of ERK phosphorylation (Figures 6(e) and 6(f)). Furthermore, the expressions of *α*-SMA, fibronectin, and collagen I in fibroblasts treated with the ERK inhibitor PD98059 were decreased (Figures 6(g) and 6(h)). These results indicate that ERK phosphorylation is increased in intestinal fibrosis and could participate in its onset and progression. Meanwhile, hucMSC-Ex could mitigate intestinal fibrosis by inhibiting ERK phosphorylation in intestinal fibroblasts.

## 4. Discussion

IBD is an autoimmune disease caused by several factors. In recent years, industrial development has been linked with an increased incidence of IBD. The treatment methods for IBD have changed with time, from mesalazine to TNF-*α* antagonists, which have greatly improved the quality of life of IBD patients [13]. However, IBD patients, especially CD patients, still suffer certain complications under the chronic inflammation that characterizes their condition, including intestinal fibrosis. Intestinal fibrosis results from repeated inflammatory stimuli and is associated with other complications of IBD, such as intestinal strictures and fistulas [14]. Although the current therapeutic methods for IBD have certain therapeutic effects, there is no therapeutic drug for intestinal fibrosis [15]. When patients develop fibrosis, tissue resection is often used to resolve the problem [16]. Therefore, researchers are eager in seeking new ideas for the treatment of intestinal fibrosis. In our study, we demonstrated that hucMSC-Ex is a new and promising treatment option for IBD-related intestinal fibrosis.

Exosomes are a class of functional small vesicles that carry abundant proteins, RNAs, DNAs, and other substances and play biological functions as signaling molecules in cell communication, registering their application in clinical trials across many diseases [17]. Exosomes are thought to play an important role in tissue repair. hucMSC-Ex could attenuate colitis by regulating macrophage pyroptosis via the miR-378a-5p/NLRP3 axis [10]. In addition, a study found that hucMSC-Ex could attenuate renal interstitial fibrosis in a rat UUO model by regulating YAP degradation through CK1*δ*/*β*-TRCP [18]. In this study, we used ultracentrifugation to successfully extract exosomes having diameters between 30 and 150 nm, vesicle-like shapes, and positive for CD81, CD9, and HSP70 as often used markers for exosome identification [19, 20]. At the same time, we used the extracted hucMSC-Ex to treat the DSS-induced mouse IBD model, where results showed that hucMSC-Ex effectively alleviated the occurrence of DSS-induced colonic fibrosis in mice, which was mainly evident in the downregulated fibrosis-related indicators in the colon, along with increased colon length in mice, decreased colon weight/length ratio, and relieved intestinal inflammation.

Fibroblasts are a type of cells in the body responsible for filling and supporting connective tissue. In the process of tissue damage repair, fibroblasts are activated under stimulation and expand. Under normal physiological conditions, after the injury part has healed, cells would undergo apoptosis and no longer play roles in tissue repair. However, when the tissue is continuously damaged, this normal pattern is disrupted and the fibroblasts continue to proliferate and expand, eventually leading to the destruction of the ECM and fibrosis occurrence. Comparing fibroblasts from colonic strictures and normal parts under the same conditions, it was found that fibroblasts from colonic strictures had a 10-fold increase in collagen production [21]. TGF-*β* is an important factor regulating the production of ECM by fibroblasts. TGF-*β* could induce the transformation of fibroblast phenotype to myofibroblast phenotype, and the process of induction is also the process of tissue fibrosis [22]. In addition to its role in fibroblast transformation, TGF-*β* also plays a role in fibroblast proliferation and migration [23]. Studies on the role of stem cell exosomes in fibroblasts have mixed results, and different cell-derived exosomes appear to play different roles in regulating fibroblast activation. hucMSC-Ex can inhibit the activation of hepatic stellate cells by activating p21-activated kinase-2 and upregulating miR-455-3p, thereby inhibiting the occurrence of liver fibrosis. In contrast, in a model of diabetic wound healing, exosomes secreted by human-induced pluripotent stem cells could promote collagen synthesis [24]. In this study, TGF-*β* was used to induce the proliferation and migration of intestinal fibroblasts CCD18-Co, and hucMSC-Ex was used for treatment. The results indicated that hucMSC-Ex inhibits the proliferation and migration of fibroblasts. Moreover, the study detected the expression levels of *α*-SMA, collagen I, collagen III, and fibronectin in fibroblasts induced by TGF-*β* and treated with hucMSC-Ex, and the results showed that hucMSC-Ex could inhibit the expression of these fibrosis indicators. We also found that different collagens respond differently to hucMSC-Ex treatment, as the inhibition of collagen I was observed after 24 hours of hucMSC-Ex treatment while the inhibition of collagen III was observed after 72 hours. Our study proves that hucMSC-Ex could inhibit TGF-*β*-induced collagen synthesis. However, a study found that exosomes released from human-induced pluripotent stem cell-derived MSCs could promote the synthesis of collagen I and collagen III [24]. The different times of exosome treatment may have different effects on the collagen product in fibroblasts, although there is little research in this area. Future studies could further explore this observation.

ERK is the final component of the Raf-MEK-ERK signaling module, which acts downstream of receptor tyrosine kinases and controls multiple cellular processes, including cell proliferation and differentiation. Activated bi-phosphorylated ERKs transmit pathway activation to cells through the phosphorylation of multiple substrates. The ERK pathway plays a regulatory role in a variety of cellular activities. Abnormalities in the ERK pathway could cause neurodevelopmental disorders. On one hand, ERK activation causes the proliferation of cancer cells. ERK has a proapoptotic effect, and enhanced ERK1/2 signaling can lead to tumor cell death [25]. In addition, an earlier study from our research team found that the inhibition of ERK phosphorylation in neutrophils alleviates DSS-induced IBD in mice [26]. Meanwhile, the role of ERK in fibrosis has also been reported. In the liver, the inactivation of the ERK pathway reduces fibrosis [27]. Regulation of the STAT3/Erk/Akt tandem pathway in the Kupffer cells can affect cell polarization and affect the development of fibrosis [28]. TGF-*β*1/IL-11/MEK/ERK signaling can mediate aging-related pulmonary fibrosis in a Bmi-1-deficient model of premature aging [29]. Therefore, we wondered whether ERK also played a role in intestinal fibrosis and whether exosomes could alleviate intestinal fibrosis through ERK. We found elevated levels of ERK phosphorylation in the fibrotic colon tissues of mice and TGF-*β*-induced fibroblasts, and hucMSC-Ex treatment reduced fibroblast-related molecules (*α*-SMA, collagen I, and fibronectin) and ERK phosphorylation. Moreover, treatment of TGF-*β*-induced fibroblasts with ERK phosphorylation inhibitors showed that the expression of *α*-SMA, collagen I, and fibronectin decreased. All the above results indicated that hucMSC-Ex could inhibit the ERK phosphorylation level in fibroblasts and thus inhibit the development of fibrosis.

This study demonstrates that exosomes can affect the occurrence of IBD-associated intestinal fibrosis by regulating ERK phosphorylation in human intestinal fibroblasts. However, our study has shortcomings, including the lack of a detailed mechanism of ERK phosphorylation in fibrosis inhibition and the use of only intestinal fibroblasts in the ERK inhibitor PD98059 experiment, which makes the evidence weak. In addition, there is a lack of data on clinical samples. There is still some gap between the simulated disease occurrence process and the real disease occurrence; thus, clinical studies are needed, which could lead to the safe use of exosomes in humans for IBD-related intestinal fibrosis treatment. Many cells play a role in the development of fibrosis. Studies have proved that hucMSC-Ex can regulate the proliferation and activation of intestinal fibroblasts. Whether other intestinal cells can also be regulated by hucMSC-Ex can also be further investigated. Nonetheless, our findings are significant and inspire further exploration in this area. We expect these findings to be linked to previous results in other organ fibrosis and IBD and provide new insights for inhibiting and reversing fibrosis.

## 5. Conclusion

hucMSC-Ex alleviates DSS-induced IBD-related intestinal fibrosis in mice by inhibiting TGF-*β*-induced intestinal fibroblast proliferation, migration, and activation. The hucMSC-Ex relieve-effect is associated with its inhibition of ERK phosphorylation in intestinal fibroblasts, leading to reduced expression of fibrotic molecules to alleviate intestinal fibrosis.

## Figures and Tables

**Table 1:** DAI scores.

Weight loss (%)	Stool shape	Bleeding in stool	Scores
0	Normal	None	0
1~5	Loose	Occult blood positive	1
6~10			2
11~15	Watery	Gross bloody stool	3
>16			4

**Table 2:** cDNA synthesis system.

Reagent	Volume
5x gDNA wiper mix	2 *μ*l
10x RT mix	2 *μ*l
HiScript III enzyme mix	2 *μ*l
Oligo (dT)_20_VN	1 *μ*l
Random hexamers	1 *μ*l
RNase-free H_2_O	12 *μ*l

**Table 3:** QRT-PCR reaction system.

Reagent	Volume
SYBR green mix	10 *μ*l
ddH_2_O	4.2 *μ*l
Forward primer	0.4 *μ*l
Reverse primer	0.4 *μ*l
Spermine	3 *μ*l

**Table 4:** Primer sequences.

Species	Genes	Sequences	Annealing temperature
Mice	*β*-Actin	F: TCCTCCTGAGCGCAAGTACTCT	58°C
		R: GCTCAGTAACAGTCCGCCTAGAA	
	TGF-*β*	F: CCAGATCCTGTCCAAACTAAGG	58°C
		R: CTCTTTAGCATAGTAGTCCGCT	
	*α*-SMA	F: GCGGTGGCTATTCCTTCGTGACTAC	56°C
		R: CGTCAGGCAGTTCGTAGCTCTTC	
	COL1A1	F: TGAACGTGGTGTACAAGGTC	58°C
		R: CCATCTTTACCAGGAGAACCAT	
	COL3A1	F: AGTCGGAGGAATGGGTGGCTATC	58°C
		R: CAGGAGATCCAGGATGTCCAGAGG	
	FN1	F: CTATAGGATTGGAGACACGTGG	58°C
		R: CTGAAGCACTTTGTAGAGCATG	

Human	*β*-Actin	F: TCCTCCTGAGCGCAAGTACTCT	58°C
		R: GCTCAGTAACAGTCCGCCTAGAA	
	*α*-SMA	F: TCGTGCTGGACTCTGGAGATGG	58°C
		R: CCACGCTCAGTCAGGATCTTCATG	
	COL1A1	F: AAAGATGGACTCAACGGTCTC	60°C
		R: CATCGTGAGCCTTCTCTTGAG	
	COL3A1	F: GGCTACTTCTCGCTCTGCTTCATC	58°C
		R: TCTCTATCCGCATAGGACTGACCAAG	
	FN1	F: AATAGATGCAACGATCAGGACA	58°C
		R: GCAGGTTTCCTCGATTATCCTT	

## Supplementary Materials

Supplementary Materials Sup.1: schematic diagram of experimental design. (A) Schematic diagram of animal model; (B) cell model of hucMSC-Ex treatment; (C) cell model of PD98059 treatment.
