# Supplementary material for: hucMSC-Ex Alleviates IBD-Associated Intestinal Fibrosis by Inhibiting ERK Phosphorylation in Intestinal Fibroblasts
Source: Stem Cells Int. 2023 Feb 17;2023:2828981. doi: 10.1155/2023/2828981 (PMC9957621; doi:10.1155/2023/2828981)
Supplement: Supplementary Materials — Sup.1: schematic diagram of experimental design. (A) Schematic diagram of animal model; (B) cell model of hucMSC-Ex treatment; (C) cell model of PD98059 treatment. [file 2828981.f1.docx]

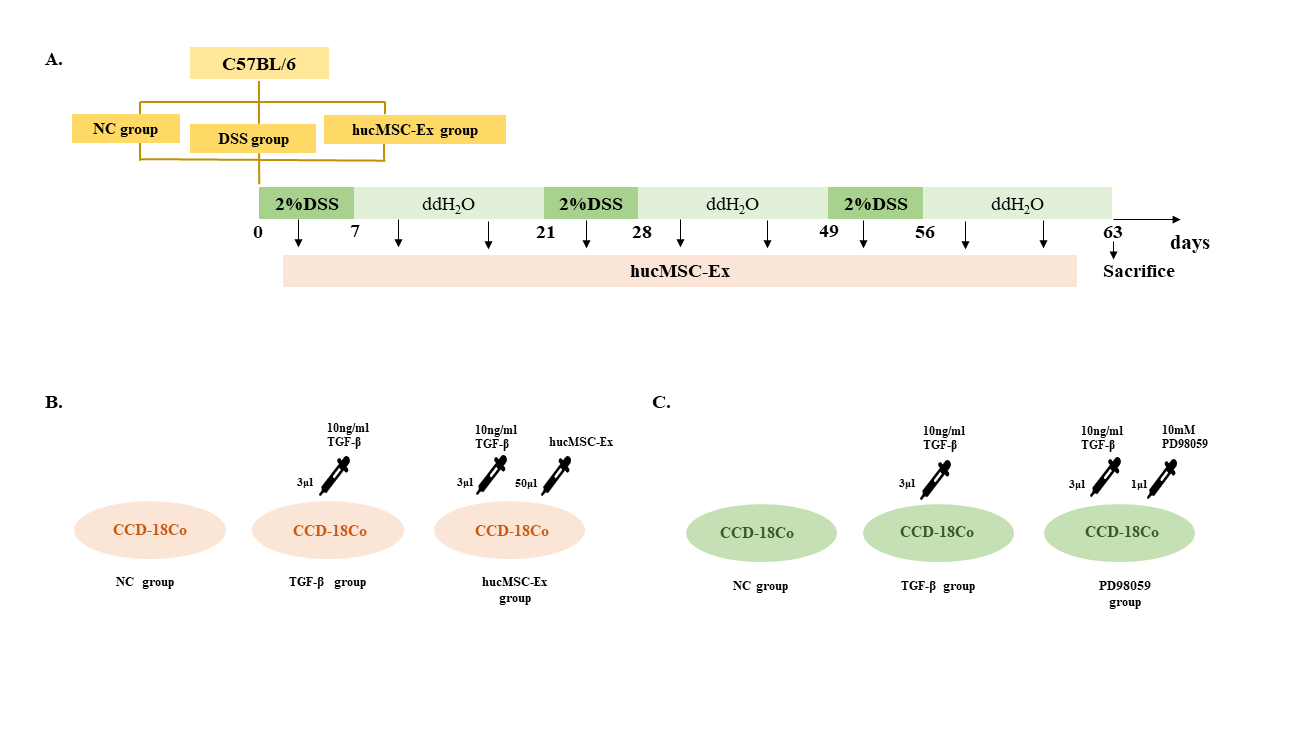
 **Sup.1. Schematic diagram of experimental design. (A) Schematic diagram of animal model; (B) Cell model of hucMSC-Ex treatment; (C) Cell model of PD98059 treatment.**
